# Filtering sequencing artifacts from FFPE tissues: an orientation-bias-based statistical tool

**DOI:** 10.1093/bib/bbaf631.054

**Published:** 2025-12-12

**Authors:** Moyukh S Khan, Davd J H Shih

## Abstract

Formalin-fixed paraffin-embedded (FFPE) tissue preservation, while cost-effective and stable in ambient conditions, induces DNA damage manifesting as C:G > T:A transition artifacts through cytosine deamination. These artifacts can dominate low-frequency variant calls and obscure true somatic mutations, which are critical for clinical interpretation. Here, we present MOBSNVF, a statistical tool to distinguish genuine variants from FFPE-induced artifacts by leveraging orientation bias, which is the preferential appearance of artifactual variants in Read 1 versus Read 2 during paired-end sequencing due to single-strand DNA damage. We comprehensively evaluated MOBSNVF against established FFPE artifact filters using simulated data spanning varying tumor purities, damage levels, and sequencing depths, as well as four real clinical datasets with matched FFPE and fresh-frozen samples. MOBSNVF achieved near-perfect classification on simulated data with an AUROC and AUPRC of 0.999, maintaining robust performance across all tested conditions. On clinical samples, MOBSNVF consistently delivered superior precision-recall trade-offs and exhibited greater AUROC and AUPRC, effectively correcting the characteristic C:G > T:A mutational skew in FFPE samples while preserving non-artifactual mutation contexts. Our tool also demonstrates broad applicability to any paired-end sequencing data while extending to other single-strand lesions, including oxidative damage, thereby establishing MOBSNVF as a robust component for clinical and research sequencing workflows requiring accurate somatic variant detection from FFPE specimens.

